# The Chest Radiographic Thoracic Area Can Serve as a Prediction Marker for Morbidity and Mortality in Infants With Congenital Diaphragmatic Hernia

**DOI:** 10.3389/fped.2021.740941

**Published:** 2021-12-23

**Authors:** Meike Weis, Sosan Burhany, Alba Perez Ortiz, Oliver Nowak, Svetlana Hetjens, Katrin Zahn, Stefan Schoenberg, Thomas Schaible, Neysan Rafat

**Affiliations:** ^1^Department of Clinical Radiology and Nuclear Medicine, University Medical Center Mannheim, University of Heidelberg, Mannheim, Germany; ^2^Department of Neonatology, University Children's Hospital Mannheim, University of Heidelberg, Mannheim, Germany; ^3^Department of Obstetrics and Gynecology, University Medical Center Mannheim, University of Heidelberg, Mannheim, Germany; ^4^Department of Biomathematics and Medical Statistics, Medical Faculty Mannheim, University of Heidelberg, Mannheim, Germany; ^5^Department of Pediatric Surgery, University Children's Hospital Mannheim, University of Heidelberg, Mannheim, Germany

**Keywords:** congenital diaphragmatic hernia (CDH), survival, chronic lung disease (CLD), extracorporeal membrane oxygenation, O/E LHR, neonate

## Abstract

**Objective:** Valid postnatal prediction parameters for neonates with congenital diaphragmatic hernia (CDH) are lacking, but recently, the chest radiographic thoracic area (CRTA) was proposed to predict survival with high sensitivity. Here, we evaluated whether the CRTA correlated with morbidity and mortality in neonates with CDH and was able to predict these with higher sensitivity and specificity than prenatal observed-to-expected (O/E) lung-to-head ratio (LHR).

**Methods:** In this retrospective cohort study, all neonates with CDH admitted to our institution between 2013 and 2019 were included. The CRTA was measured using the software Horos (V. 3.3.5) and compared with O/E LHR diagnosed by fetal ultrasonography in relation to outcome parameters including survival, extracorporeal membrane oxygenation (ECMO) support, and chronic lung disease (CLD).

**Results:** In this study 255 neonates were included with a survival to discharge of 84%, ECMO support in 46%, and 56% developing a CLD. Multiple regression analysis demonstrated that the CRTA correlates significantly with survival (*p* = 0.001), ECMO support (*p* < 0.0001), and development of CLD (*p* = 0.0193). The CRTA displayed a higher prognostic validity for survival [area under the curve (AUC) = 0.822], ECMO support (AUC = 0.802), and developing a CLD (AUC = 0.855) compared with the O/E LHR.

**Conclusions:** Our data suggest that the postnatal CRTA might be a better prognostic parameter for morbidity and mortality than the prenatal O/E LHR.

## Introduction

Congenital diaphragmatic hernia (CDH) is characterized by failure of diaphragmatic development and thoracic herniation of abdominal organs, leading to lung hypoplasia and persistent pulmonary hypertension of the newborn (PPHN) (1). The clinical course of this malformation is variable, wherefore a reliable estimation of prognosis is of particular importance. As lung hypoplasia, besides PPHN, is an important determinant marker for mortality and morbidity in CDH patients (2), quantification of the absolute lung volume is a tool to predict prognosis (3–5).

In the past years, numerous parameters have been evaluated to predict prognosis in CDH patients. The observed-to-expected (O/E) lung-to-head ratio (LHR) diagnosed by fetal ultrasonography shows a high prognostic validity for clinical outcome (6–9). Although measuring O/E LHR has been established as standard approach, this parameter can be utilized only in patients with prenatal diagnosis of CDH. For this reason, it would be preferable to also establish a parameter for prognosis, which is dependent only on postnatal data. Estimation of the chest radiographic thoracic area (CRTA) is an alternative to assess lung volume on a chest radiograph (10, 11). Recently, it has been demonstrated that CRTA can predict survival in infants with severe CDH with high sensitivity and moderate specificity (12).

In this study, we evaluated whether the postnatal CRTA was able to predict morbidity and mortality with higher sensitivity and specificity than prenatal O/E LHR. As main morbidity outcome parameters, extracorporeal membrane oxygenation (ECMO) support and chronic lung disease (CLD) were selected.

## Materials and Methods

### Subjects and Clinical Data

All newborn infants with CDH treated between January 1, 2013, and December 31, 2019, at our neonatal intensive care unit at the Department of Neonatology of the University Children's Hospital Mannheim, University of Heidelberg, were included in this retrospective study. Exclusion criteria were outborn patients; preterm infants <34 weeks' gestational age; patients with associated anomalies, syndromes, or chromosomal aberrations; newborns who died shortly after birth due to a different complication; and patients for which an initial radiograph was missing (Supplementary Figure 1).

Demographic, prenatal and perinatal, and clinical data were collected from the patient's records. Estimation of CDH disease severity was based on prenatal diagnostic measures including fetal ultrasonography for liver position and measurement of observed to expected (O/E) LHR (13). The diagnosis of CLD was made as reported before (9, 14): if there was an additional need for oxygen supplementation at day 28 after birth, CLD was diagnosed. Severity of CLD was differentiated into three grades according to the additional need for oxygenation at day 56 after birth: mild CLD with no need for supplemental inspired oxygen (fraction of inspired oxygen [Fio_2_] ≤ 0.21), moderate CLD (Fio_2_, 0.22–0.29), and severe CLD (Fio_2_ ≥ 0.30). This study was approved by the local ethics committee of the Medical Faculty Mannheim of the University of Heidelberg (reference no. 2020-802R).

### Chest Radiographic Thoracic Area

**Figure 1:**
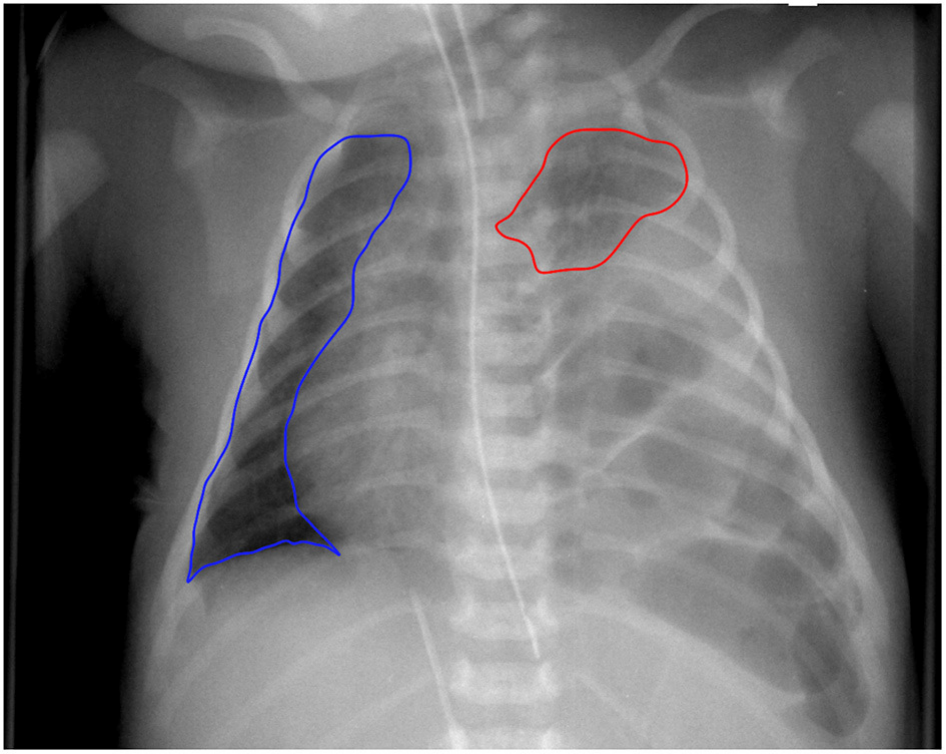
Delineation of chest radiographic thoracic area. Example of a newborn with left-sided congenital diaphragmatic hernia. CRTA was calculated as the sum of area of ipsilateral and contralateral lung. Segmentation was performed manually.

To assess the CRTA, the first preoperative chest radiograph of the neonates with CDH in the first 24 h after birth was included in the analysis. Chest x-rays were obtained in supine position with the tube 1 m above the patient. Lung borders were delineated manually using dedicated software (Horos, V. 3.3.5; Nimble Co. LLC d/b/a Purview in Annapolis, MD, USA) and a freehand tool, applying the following criteria (Figure 1): (a) contralateral: following border of the heart → delineation of the diaphragm → delineation of the thoracic wall → delineation of the upper mediastinum; (b) ipsilateral: delineation of hypoplastic lung. Areas of contralateral and ipsilateral lung were summed up to calculated CRTA. Correction of magnification error was performed by division with the factor 1.04.

### Statistical Analysis

Statistical analysis was performed with SAS® version 9.4 (SAS Institute Inc., USA). Descriptive statistics were used to describe the demographic characteristics of the patients and to analyze the distribution of survival, ECMO support, and development of CLD. For metric variables, position and scatter measures, as well as distribution, were calculated. Nominal and ordinal variables were characterized using absolute and relative frequencies. The distribution of the variables was verified using the Shapiro-Wilk test. Inductive statistics was applied to evaluate the observed relationships and differences from descriptive statistics for their statistical significance. Differences in a metric characteristic between two unrelated groups were examined using the Mann–Whitney *U* test. For nominal or ordinal characteristics, the χ^2^ test or Fisher exact test was used accordingly. In order to analyze the correlation between two metric variables, a correlation analysis according to Pearson was carried out for normally distributed data and a correlation analysis according to Spearman for non–normally distributed data. The influence of various independent parameters on survival, ECMO support, and development of CLD was analyzed in simple regression models. Subsequently, by means of step-by-step selection, significant variables were included in a multiple regression model. With the parameters O/E LHR and CRTA, logistic regression models were prepared for the respective endpoints. The significance of the parameters was assessed with receiver operating characteristic (ROC) analysis by calculating the area under the curve (AUC). A cutoff value was determined using the Youden index. In a final step, the correlation of the O/E LHR or CRTA was evaluated with the duration of hospitalization or the duration of mechanical ventilation. Linear regression models were created, and their quality was evaluated by calculating coefficients of determination. *p* ≤ 0.05 was considered significant.

## Results

### Demographic and Clinical Characteristics of the Study Cohort

Between January 2013 and December 2019, 403 neonates with CDH were treated at our center, of whom 255 neonates were included in this study. For an overview of the recruitment of CDH patients into this study and the characteristics of the dropouts, see Supplementary Figure 1. Of the included study population, 40 patients were transferred to hospitals in the patient's home area after stabilization, surgical repair, and successful weaning. In these patients, the entire duration of the inpatient treatment could not be assessed. For an overview of the characteristics of the study population, refer to Table 1.

**Table 1:** Patient characteristic of the study population (*n* = 255).

Prenatal data	
Left-sided defect, n (%)	225 (88)
Liver herniation, n (%)	159 (65)
LHR	1.60 (0.5–4.6)
O/E LHR, %	38 (21–83)
rLV, %	33.5 (9–94.4)
FETO, n (%)	7 (3)
Demographics and birth	
Male sex, n (%)	145 (57)
GA, weeks	38 (34–40.3)
Birth weight, g	3,030 (1,420–4,600)
Apgar score after 5 min	8 (0–10)
CRTA, mm^2^	1,155 (254–3,225)
Ventilation and additive therapies
oxygenation index	13.9 (1.1–83.3)
HFOV, n (%)	104 (41)
HFOV on day 1, n (%)	63 (25)
Duration of mechanical ventilation, days	19.1 (0.1–214)
iNO, n (%)	178 (70)
iNO on day 1, n (%)	154 (63)
ECMO, n (%)	118 (46)
Duration of ECMO, days	9 (1.1–20.1)
Surgical	
Operated, n (%)	229 (98)
Patch repair, n (%)	189 (83)
Days to full enteral feeding	25 (0.1–178)
Outcome	
Days of hospitalization	42 (0.1–391)
Discharge with oxygen therapy (Fio_2_ >0.21), n (%)	1 (1)
Discharge with HFNC, n (%)	5 (3)
Discharge with home mechanical ventilation, n (%)	4 (2)
CLD, n (%)	117 (56)
Mild, n	78 (75)
Moderate, n	17 (16)
Severe, n	9 (9)
Survival to discharge, n (%)	213 (84)

*Data are expressed as median (interquartile range) or relative frequency (%)*.

*CLD, chronic lung disease; CRTA, chest radiographic thoracic area; ECMO, extracorporeal membrane oxygenation; FETO, fetoscopic endoluminal tracheoscopic occlusion; HFOV, high-frequency oscillation ventilation; iNO, inhaled nitric oxide; LHR, lung-to-head ratio; O/E, observed-to-expected; rLV, relative lung volume*.

### Correlation of Clinical Parameters With Survival, ECMO Support, and CLD

We first correlated several major clinical parameters with survival, ECMO support, and CLD in our study population. The neonates who died showed prenatally a significant lower O/E LHR and a lower relative lung volume (rLV) compared with the survivors (Table 2). They also revealed more frequently a liver herniation and received more often fetoscopic endoluminal tracheal occlusion (FETO) (Table 2). On day 1 of life and thereafter, a treatment with HFOV, inhaled nitric oxide (iNO), or ECMO was significantly more often established in this population (Table 2).

**Table 2:** Comparison of patient characteristics in relation to survival.

	**Survived (*n* = 213)**	**Deceased (*n* = 42)**	***p*-value**
**Prenatal data**			
Left-sided defect	87% (186/213)	93% (39/42)	0.434
Liver herniation	61% (126/208)	87% (33/38)	0.002
LHR	1.7 (0.5–4.6)	1.4 (0.9–3.2)	0.002
rLV, %	35 (18–94.4)	24.2 (9–50)	<0.0001
FETO	1% (3/212)	10% (4/42)	0.016
**Demographics and birth**			
Male sex	58% (124/213)	50% (21/42)	0.326
GA, weeks	38 (34–40.3)	38 (34–39.6)	0.119
Birth weight, g	3,100 (2,000–4,600)	2,855 (1,420–3,500)	0.004
Apgar score after 5 min	8 (4–10)	7 (0–9)	<0.0001
**Ventilation and additive therapies**			
Oxygenation index	9.4 (1.1–83.3)	24.4 (4.1–54.5)	<0.0001
HFOV	33% (70/213)	81% (34/42)	<0.0001
HFOV on day 1	19% (39/210)	60% (24/40)	<0.0001
iNO	65% (138/213)	95% (40/42)	<0.0001
iNO on day 1	58% (119/205)	90% (35/39)	0.0002
ECMO	39% (84/213)	81% (34/42)	<0.0001

*Data are expressed as median (interquartile range) or relative frequency (%)*.

*ECMO, extracorporeal membrane oxygenation; FETO, fetoscopic endoluminal tracheoscopic occlusion; GA, gestational age; HFOV, high-frequency oscillation ventilation; iNO, inhaled nitric oxide; LHR, lung-to-head ratio; rLV, relative lung volume*.

The patient population requiring ECMO support also showed prenatally a significant lower O/E LHR and a lower rLV prenatally compared with the patients without ECMO support (Table 3). In this patient population, a left-sided CDH and a liver herniation were more frequently present (Table 3). Also, in this population on day 1 of life and thereafter, a treatment with HFOV, iNO, or ECMO was significantly more often established (Table 3).

**Table 3:** Comparison of patient characteristics in relation to ECMO support.

	**No ECMO (*n* = 137)**	**ECMO (*n* = 118)**	***p*-value**
**Prenatal data**			
Left-sided defect	95% (130/137)	81% (95/118)	0.0004
Liver herniation	43% (66/131)	90% (103/115)	<0.0001
LHR	1.9 (1.1–4.6)	1.4 (0.5–3.2)	<0.0001
rLV, %	38.3 (20–94.4)	28.6 (9–84.1)	<0.0001
FETO	1% (1/136)	5% (6/118)	0.052
**Demographics and birth**			
Male sex	55% (75/137)	59% (70/118)	0.462
GA, weeks	38.1 (34.7–40.3)	38 (34–39.9)	0.1
Birth weight, g	3,100 (1,420–4,375)	2,960 (1,800–4,600)	0.502
Apgar score after 5 min	8 (0–10)	8 (4–10)	<0.0001
**Ventilation and additive therapies**			
Oxygenation index	5.4 (1.5–83.3)	21.6 (1.1–83.3)	<0.0001
HFOV	18% (25/137)	67% (79/118)	<0.0001
HFOV on day 1	11% (15/135)	42% (48/115)	<0.0001
iNO	44% (60/137)	100% (118/118)	<0.0001
iNO on day 1	38% (60/137)	100% (118/118)	<0.0001

*Data are expressed as median (interquartile range) or relative frequency (%)*.

*ECMO, extracorporeal membrane oxygenation; FETO, fetoscopic endoluminal tracheoscopic occlusion; GA, gestational age; HFOV, high-frequency oscillation ventilation; iNO, inhaled nitric oxide; LHR, lung-to-head ratio; rLV, relative lung volume*.

When comparing patients with and without CLD, patients with CLD showed prenatally a significant lower O/E LHR and a lower rLV prenatally compared with the patients without CLD (Table 4). Also, in this patient population, a left-sided CDH and a liver herniation were more frequently present (Table 4). In patients with CLD on day 1 of life and thereafter, a treatment with HFOV or iNO was significantly more often established (Table 4).

**Table 4:** Comparison of patient characteristics in relation to CLD.

	**No CLD (*n* = 91)**	**CLD (*n* = 117)**	***p*-value**
**Prenatal data**			
Left-sided defect	92% (84/91)	83% (97/117)	0.454
Liver herniation	38% (34/89)	83% (95/115)	<0.0001
LHR	2.0 (1.1–3.5)	1.5 (0.5–3.2)	<0.0001
rLV, %	41 (22.4–94.4)	30.7 (18–65)	<0.0001
FETO	0% (0/91)	3% (4/117)	0.133
**Demographics and birth**			
Male sex	59% (54/91)	58% (68/117)	0.859
GA, weeks	38.1 (34.9–40.3)	38 (34–40.1)	0.155
Birth weight, g	3,120 (2,000–4,400)	2,960 (2,050–4,600)	0.084
Apgar score after 5 min	8 (4–10)	8 (4–10)	<0.0001
**Ventilation and additive therapies**			
Oxygenation index	3.6 (1.5–30)	17.9 (1.1–83.3)	<0.0001
HFOV	15% (14/91)	51% (60/117)	<0.0001
HFOV on day 1	9% (8/90)	27% (31/115)	0.0011
iNO	32% (29/91)	92% (109/117)	<0.0001
iNO on day 1	27% (24/90)	86% (94/110)	<0.0001
ECMO	6% (5/91)	72% (84/117)	<0.0001

*Data are expressed as median (interquartile range) or relative frequency (%)*.

*CLD, chronic lung disease; ECMO, extracorporeal membrane oxygenation; FETO, fetoscopic endoluminal tracheoscopic occlusion; GA, gestational age; HFOV, high-frequency oscillation ventilation; iNO, inhaled nitric oxide; LHR, lung-to-head ratio; rLV, relative lung volume*.

### Correlation of O/E LHR With CRTA

**Figure 2:**
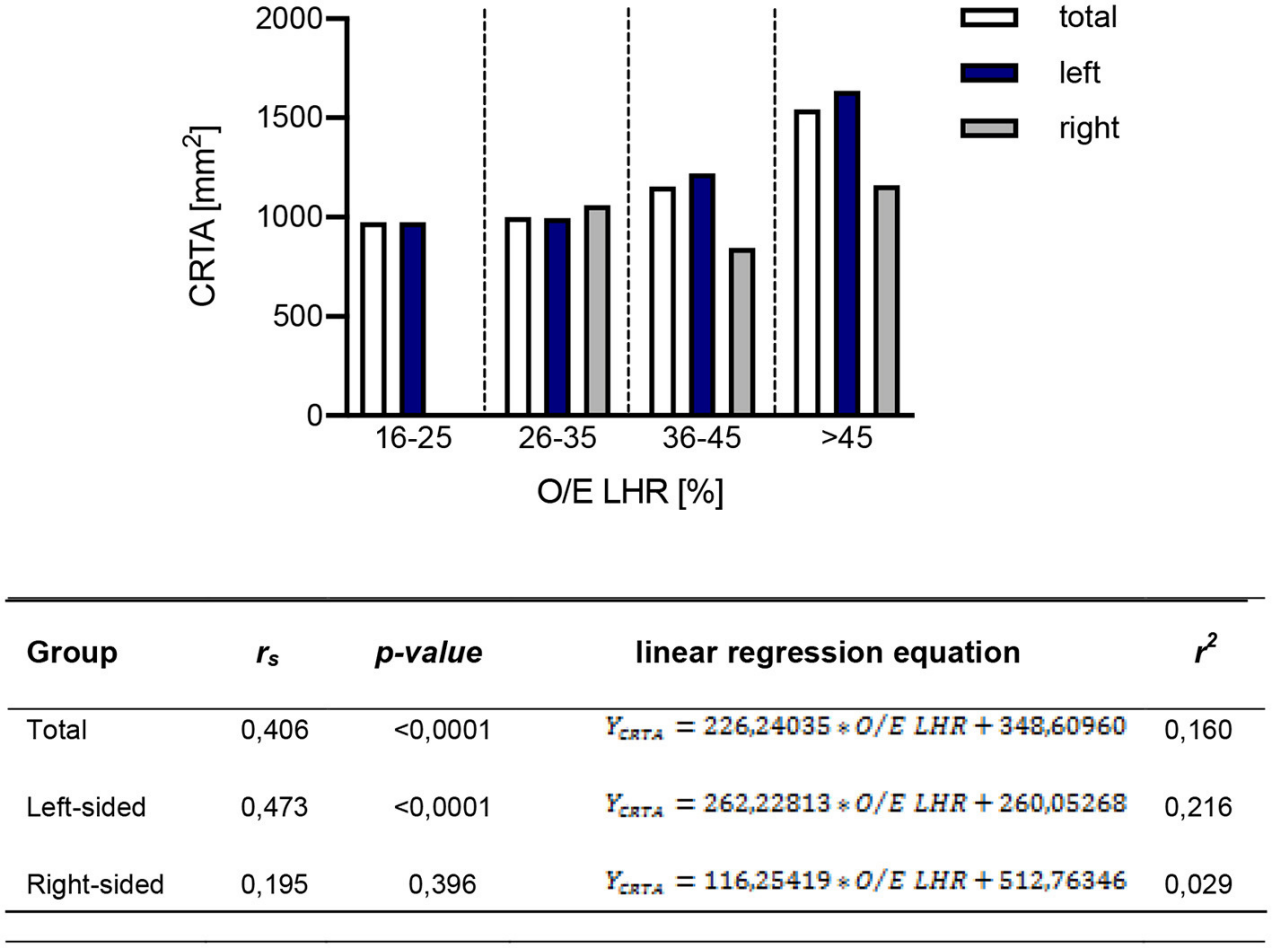
Correlation of observed-to-expected lung-to-head ratio with CRTA. The correlation of observed-to-expected lung-to-head ratio and CRTA is shown in the total study population [*r*_s_(168) = 0.406, *p* < 0.0001], in the group of left-sided [*r*_s_(147) = 0.473, *p* < 0.0001] and right-sided CDH [*r*_s_(21) = 0.195, *p* = 0.396]. CRTA, chest radiographic thoracic area; LHR, lung-to-head ratio; O/E, observed-to-expected; *r*_s_, correlation coefficient; *r*^2^, determination coefficient.

The correlation of O/E LHR and CRTA was assessed by Spearman correlation analysis. In the total study population [*r*_s_(168) = 0.406, *p* < 0.0001] and in the population of patients with left-sided CDH [*r*_s_(147) = 0.473, *p* < 0.0001], a significant correlation could be demonstrated for both parameters. Patients with right-sided CDH showed no significant correlation with the two parameters [*r*_s_(21) = 0.195, *p* = 0.396; Figure 2]. The resulting values from the linear regression analysis to display the correlation of O/E LHR and CRTA are shown in Figure 2.

### Correlation of Independent Clinical Parameters With CRTA

**Figure 3:**
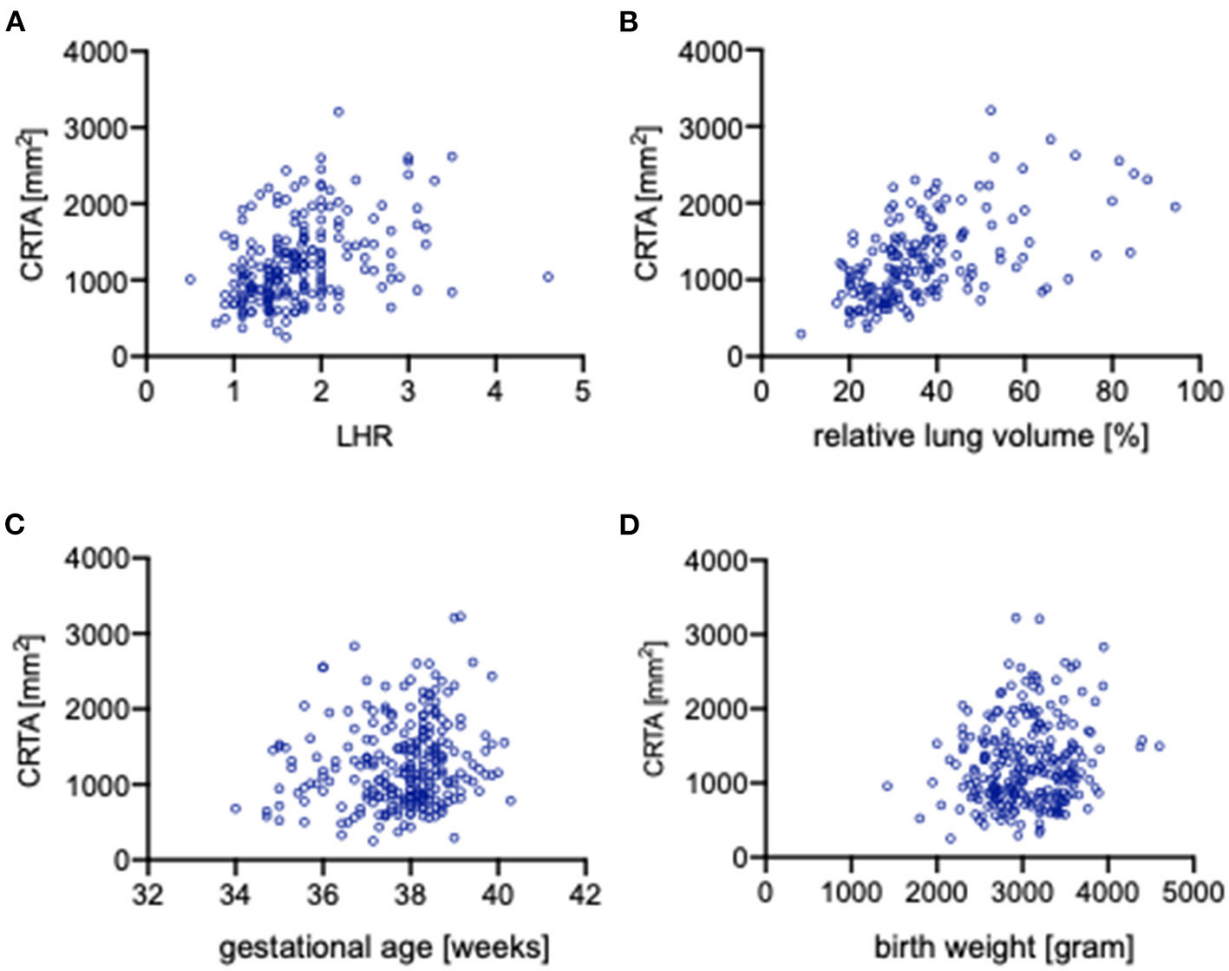
Correlation of independent clinical parameters with CRTA. Scatterplots portraying the correlation of CRTA to **(A)** lung-to-head ratio (*r* = 0.388, *p* < 0.0001), **(B)** relative lung volume (*r* = 0.544, *p* < 0.0001), **(C)** gestational age (*r* = 0.101, *p* = 0.107), and **(D)** birth weight (*r* = 0.15, *p* = 0.017). CRTA, chest radiographic thoracic area; LHR, lung-to-head ratio.

The impact of independent clinical parameter on the CRTA was analyzed by correlation analysis. O/E LHR (*r* = 0.388, *p* < 0.0001; Figure 3A), rLV (*r* = 0.544, *p* < 0.0001; Figure 3B), and birth weight (*r* = 0.150, *p* = 0.017; Figure 3) correlate significantly with CRTA, but not gestational age (*r* = 0.101, *p* = 0.107; Figure 3C). Neonates that were treated with high-frequency oscillation on day 1 of life had a significantly lower CRTA (929 mm^2^ [254–2,832]) compared with neonates treated only with conventional ventilation therapy (1,266 [374–3,225], *p* < 0.0001) (data not shown).

### Prognostic Value of O/E LHR and CRTA for Survival

**Figure 4:**
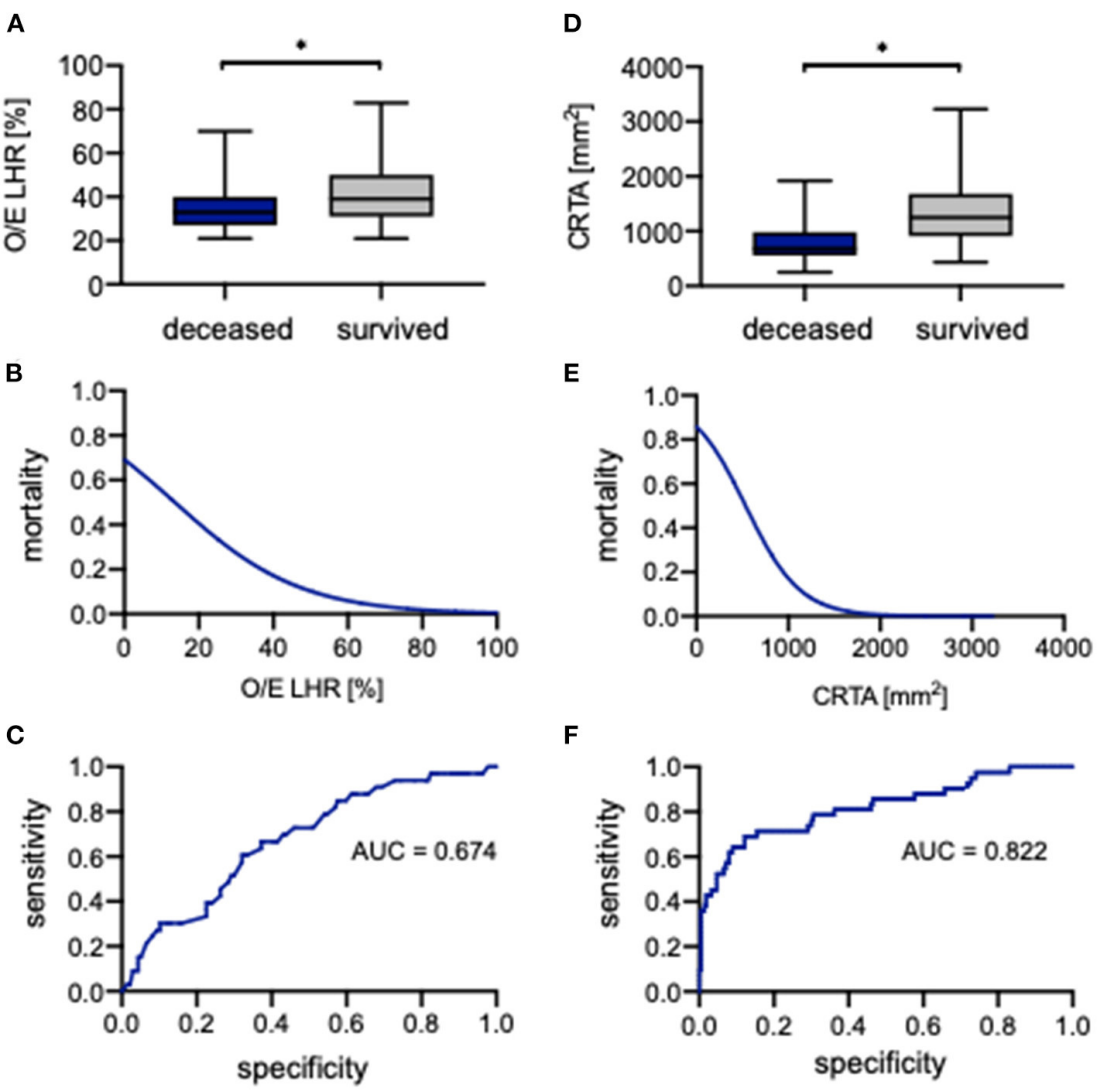
Correlation of observed-to-expected lung-to-head ratio and CRTA to survival. Boxplots of **(A)** observed-to-expected lung-to-head ratio and **(D)** CRTA are exhibited for deceased and surviving infants (whiskers are shown as minimum and maximum). Also shown is the logistical regression **(B,E)** and ROC curve **(C,F)** of observed-to-expected lung-to-head ratio and CRTA for the prediction of mortality in the study population. *Significant difference in the *U* test. AUC, area under curve; CRTA, chest radiographic thoracic area; LHR, lung-to-head ratio; O/E, observed-to-expected.

The evaluation of O/E LHR between deceased and surviving neonates showed a significant difference (33.0% [21.0%−70.0%] vs. 39.0% [21.0%−83.0%], *p* = 0.002; Figure 4A). To assess the association of O/E LHR with mortality, a logistical regression analysis was performed, and the following regression equation describes the mortality rate depending on O/E LHR:


$$\begin{array}{l} P_{mortality}=\frac{e^{\left(0.8081-0.05943^{*}O/E\;LHR\right)}}{1+e^{\left(0.8081-0.05943^{*}O/E\;LHR\right)}} \end{array}$$

An increasing O/E LHR is associated with lower mortality (Figure 4B). Therefore, the probability of neonates to die with an O/E LHR of 60% is 5%, whereas the probability to die with an O/E LHR of 15% is 45%. The ROC curve to predict survival showed an AUC of 0.674 (Figure 4C). When using 36% as a cutoff, the O/E LHR had a sensitivity of 63% and a specificity of 67% for the prediction of mortality.

Also, for CRTA, a significant difference was revealed between deceased and surviving neonates (682 mm^2^ [254–1,919 mm^2^] vs. 1,252 mm^2^ [434–3,225 mm^2^], *p* < 0.0001; Figure 4D). The association of CRTA and mortality could be described by the following regression equation:


$$\begin{array}{l} P_{mortality}=\frac{e^{\left(1.807-0.003376^{*}CRTA\right)}}{1+e^{\left(1.807-0.003376^{*}CRTA\right)}} \end{array}$$

Consistent with O/E LHR, an increasing CRTA is also associated with lower mortality (Figure 4E); for example, the probability to die with a CRTA of 1,800 mm^2^ is <1%, whereas the probability to die with a CRTA of 200 mm^2^ is 75%. The ROC curve indicated an AUC of 0.822 (Figure 4F). When using 806 mm^2^ as a cutoff, the CRTA had a sensitivity of 88% and a specificity of 69% for the prediction of mortality.

In the univariate analysis, the parameters O/E LHR, CRTA, LHR, rLV, liver herniation, FETO, birth weight, 5-min Apgar, HFOV, HFOV on day 1, iNO, iNO on day 1, oxygenation index, and ECMO were identified to be statistically significant for survival. The parameters oxygenation index, 5-min Apgar, CRTA, and FETO were gradually introduced into a multiple logistic regression model, and it could be demonstrated that CRTA (*p* = 0.001) and 5-min Apgar score (*p* = 0.019) were independently associated with survival (AUC = 0.895) (data not shown).

To evaluate whether CRTA plays an important role in certain patient populations, we performed subgroup analysis for left-sided (*n* = 225) and right-sided CDH (*n* = 30). The highest prognostic validity of O/E LHR for survival could be seen in left-sided CDH, whereas for CRTA, it was seen in right-sided CDH (Supplementary Table 1). Supplementary Table 1 gives an overview of the significant parameter of the univariate and multivariate analyses for survival in regard to defect side.

### Prognostic Value of O/E LHR and CRTA for ECMO Support

**Figure 5:**
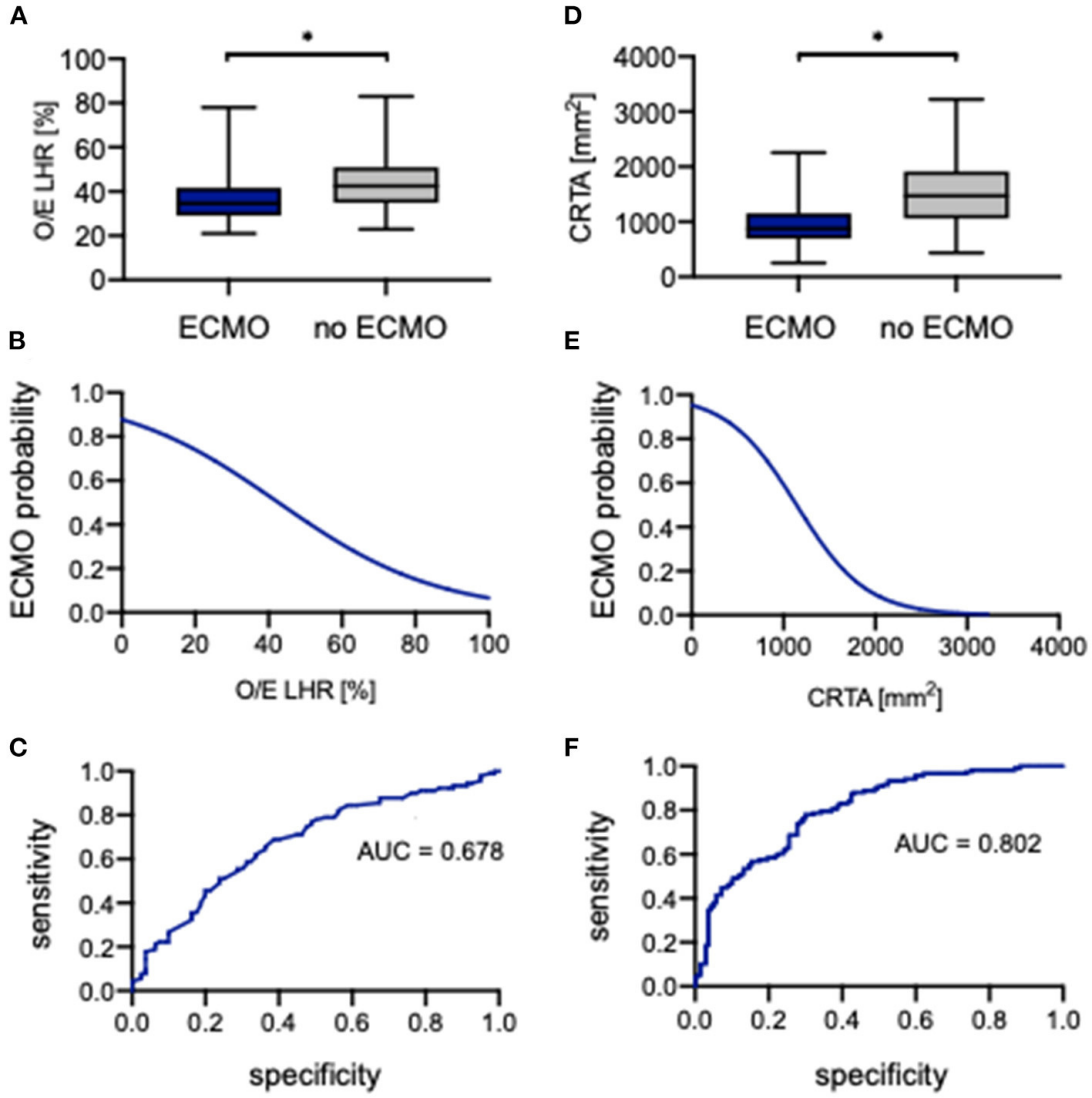
Correlation of observed-to-expected lung-to-head ratio and CRTA to ECMO. Boxplots of **(A)** observed-to-expected lung-to-head ratio and **(D)** CRTA are exhibited for patients with ECMO and without ECMO support (whiskers are shown as minimum and maximum). Also shown is the logistical regression **(B,E)** and ROC curve **(C,F)** of observed-to-expected lung-to-head ratio and CRTA for the prediction of ECMO probability in the study population. *Significant difference in the *U* test. AUC, area under curve; CRTA, chest radiographic thoracic area; ECMO, extracorporeal membrane oxygenation; LHR, lung-to-head ratio; O/E, observed-to-expected.

The evaluation of O/E LHR between neonates with ECMO support and without showed a significant difference (34.6% [21.0–78.0%] vs. 42.5% [23.0–83.0%], *p* < 0.0001; Figure 5A). The association of O/E LHR and ECMO support can be described by the following regression equation:


$$\begin{array}{l} P_{ECMO}=\frac{e^{\left(1.971-0.04615^{*}\frac{O}{E}\;LHR\right)}}{1+e^{\left(1.971-0.04615^{*}\frac{O}{E}\;LHR\right)}} \end{array}$$

An increasing O/E LHR is associated with lower ECMO support (Figure 5B). Neonates with a prenatal O/E LHR of 15% have a probability of <75% to require ECMO support, whereas neonates with an O/E LHR of 60% have a probability of <30% of cases to require ECMO support. The ROC analysis indicated an AUC of 0.678 (Figure 5C). When using 39% as a cutoff, the O/E LHR had a sensitivity of 69% and a specificity of 61% for the prediction of ECMO support.

Also, for CRTA, a significant difference was revealed between neonates with ECMO support and without (875 mm^2^ [254–2,258 mm^2^] vs. 1,470 mm^2^ [437–3,225 mm^2^], *p* < 0.0001; Figure 5D). The association of CRTA and ECMO support could be described by the following regression equation:


$$\begin{array}{l} P_{ECMO}=\frac{e^{\left(3.019-0.002642^{*}CRTA\right)}}{1+e^{\left(3.019-0.002642^{*}CRTA\right)}} \end{array}$$

Consistent with O/E LHR, an increasing CRTA is also associated with lower ECMO support (Figure 5E). Neonates with a CRTA of 2,000 mm^2^ have a probability of <10% to require ECMO support, whereas neonates with a CRTA of 600 mm^2^ have a probability of 80% to require ECMO support. The ROC curve indicated an AUC of 0.802 (Figure 5F). When using 1,188 mm^2^ as a cutoff, the CRTA had a sensitivity of 78% and a specificity of 70% for the prediction of ECMO support.

In the univariate analysis, the parameters O/E LHR, CRTA, LHR, rLV, defect side, liver herniation, 5-min Apgar, HFOV, HFOV on day 1, iNO, iNO on day 1, and oxygenation index were identified to correlate significantly with ECMO support. The parameters iNO, iNO on day 1, and oxygenation index were gradually introduced into a multiple logistic regression model, and it could be demonstrated that iNO on day 1 (*p* = 0.001) and the oxygenation index (*p* = 0.001) were independently associated with ECMO support (AUC = 0.933) (data not shown).

In the subgroup analysis in regard to defect side, the highest prognostic validity of O/E LHR for ECMO support could be seen in left-sided CDH, whereas for CRTA, it was seen in right-sided CDH (Supplementary Table 2). Supplementary Table 2 gives an overview of the significant parameters of the univariate and multivariate analysis for ECMO support in regard to defect side.

### Prognostic Value of O/E LHR and CRTA for CLD

**Figure 6:**
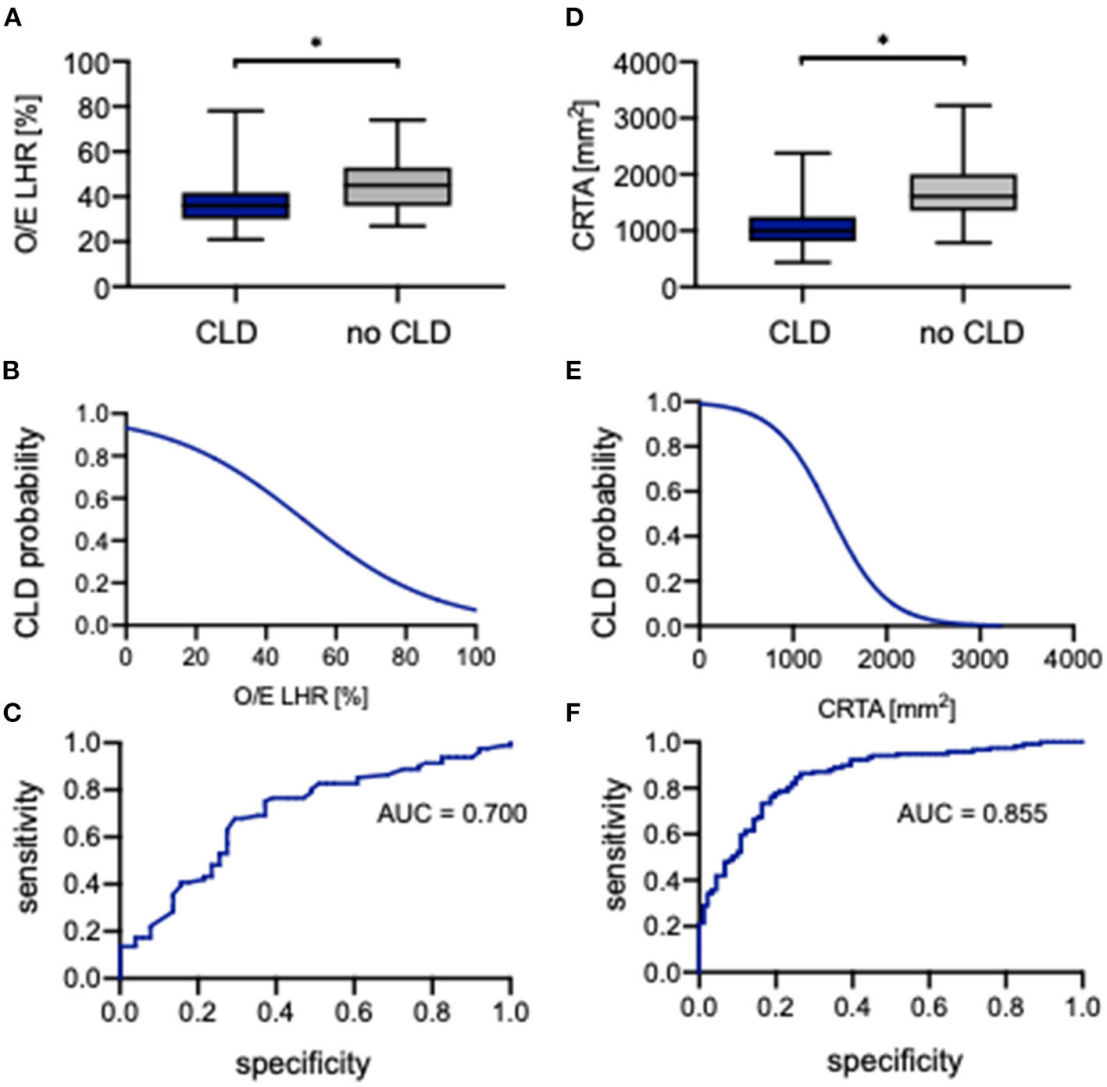
Correlation of observed-to-expected lung-to-head ratio and CRTA to CLD. Boxplots of **(A)** observed-to-expected lung-to-head ratio and **(D)** CRTA are exhibited for patients with CLD and without CLD (whiskers are shown as minimum and maximum). Also shown is the logistical regression **(B,E)** and ROC curve **(C,F)** of observed-to-expected lung-to-head ratio and CRTA for the prediction of CLD probability in the study population. *represents a significant difference in the *U* test. AUC, area under curve; CLD, chronic lung disease; CRTA, chest radiographic thoracic area; LHR, lung-to-head ratio; O/E, observed-to-expected.

The evaluation of O/E LHR between neonates with CLD and without CLD showed a significant difference (36.0% [21.0%−78.0%] vs. 45.0% [27.0%−74.0%], *p* = 0.0002; Figure 6A). The association of O/E LHR and CLD can be described by the following regression equation:


$$\begin{array}{l} P_{CLD}=\frac{e^{\left(2.626-0.05180^{*}\frac{O}{E}\;LHR\right)}}{1+e^{\left(2.626-0.05180^{*}\frac{O}{E}\;LHR\right)}} \end{array}$$

An increasing O/E LHR is associated with lower incidence of CLD (Figure 6B). Neonates with a prenatal O/E LHR of 15% have a probability of 85% to develop CLD, whereas a neonate with an O/E LHR of >60% has a probability of only 10% to develop CLD. The ROC analysis indicated an AUC of 0.700 (Figure 6C). When using 39% as a cutoff, the O/E LHR had a sensitivity of 68% and a specificity of 71% for the prediction of developing CLD.

Also for CRTA, a significant difference was revealed between neonates with CLD and without CLD (997 mm^2^ [434–2,379 mm^2^] vs. 1,610 mm^2^ [787–3,225 mm^2^], *p* < 0.0001; Figure 6D). The association of CRTA and CLD could be described by the following regression equation:


$$\begin{array}{l} P_{CLD}=\frac{e^{\left(4.653-0.003318^{*}CRTA\right)}}{1+e^{\left(4.653-0.003318^{*}CRTA\right)}}\; \end{array}$$

Consistent with O/E LHR, an increasing CRTA is also associated with lower incidence of CLD (Figure 6E). Neonates with a CRTA of 1,000 mm^2^ have a probability of 80% to develop CLD, whereas neonates with a CRTA of 2,000 mm^2^ have a probability of <10% to develop CLD. The ROC curve indicated an AUC of 0.855 (Figure 6F). When using 1,383 mm^2^ as a cutoff, the CRTA had a sensitivity of 86% and a specificity of 74% for the prediction of developing CLD. However, there was no significant association of either O/E LHR or CRTA with the severity of CLD (Supplementary Figure 2).

In the univariate analysis, the parameters O/E LHR, CRTA, LHR, rLV, defect side, liver herniation, 5-min Apgar, HFOV, HFOV on day 1, iNO, iNO on day 1, oxygenation index, and ECMO support were identified to correlate significantly with developing CLD. The parameters ECMO support, rLV, and CRTA were gradually introduced into a multiple logistic regression model, and it could be demonstrated that CRTA (*p* = 0.019), rLV (*p* = 0.009), and ECMO support (*p* = 0.003) were independently associated with developing CLD (AUC = 0.971) (data not shown).

In the subgroup analysis in regard to defect side, the highest prognostic validity of O/E LHR as well as CRTA for developing CLD could be seen in right-sided CDH (Supplementary Table 3). Supplementary Table 3 gives an overview of the significant parameters of the univariate and multivariate analyses for developing CLD in regard to defect side.

### Prognostic Value of O/E LHR and CRTA for Other Clinical Parameters

Different other clinical parameters were analyzed for their prognostic validity. In the subgroup of surviving neonates, a negative correlation of O/E LHR (*r*_s_ = −0.438, *p* < 0.0001; Supplementary Figure 3A) and CRTA (*r* = −0.352, *p* < 0.0001; Supplementary Figure 3B) could be demonstrated with the duration of mechanical ventilation. In the same subgroup, a negative correlation of O/E LHR (*r*_s_ = −0.409, *p* < 0.0001; Supplementary Figure 3C) and CRTA (*r* = −0.465, *p* < 0.0001; Supplementary Figure 3D) could be demonstrated with the duration of hospitalization.

## Discussion

The postnatally measured CRTA can predict important outcome parameters—survival, ECMO requirement, and the development of CLD—in children with CDH. In direct comparison to the established and prenatal parameter O/E LHR, it seems superior. The CRTA has initially been described by Dimitriou et al. (10) as a parameter that correlates well with functional lung parameters, such as the functional residual capacity.

Concerning survival, our results are in good agreement with previously published studies. Dassios et al. (12) demonstrated in a smaller patient cohort (*n* = 84) also an excellent prognostic accuracy of the CRTA in predicting mortality. Most recently, Amodeo et al. (15) also reported a good prognostic accuracy of the CRTA concerning survival. In contrast to results quoted before, Dimitriou et al. only found a weak performance of the CRTA in the prediction of poor outcome (11). In their study, only the postoperatively measured CRTA was associated with poor outcome and not—as used in the present study—the preoperatively quantified CRTA (11). One explanation can be the relatively small (*n* = 25) study cohort, in which also premature neonates (<34 gestational weeks) were included.

To our knowledge, there are no other studies evaluating the prognostic accuracy of the CRTA regarding ECMO requirement. Our study demonstrates an excellent prognostic accuracy for this endpoint. As ECMO therapy is available only in specialized centers, the postnatally measured CRTA can help to assess whether an outborn child should be transported into a tertiary care center.

To date, there have been few studies on the value of postnatal parameters in predicting the development of CLD in neonates with CDH. In the work of Dimitriou et al. (11), there was no correlation between preoperative CRTA and the development of CLD in CDH. A recently published study by Amodeo et al. (15) showed a significant correlation between CRTA and long-term lung function morbidity in the follow-up of neonates with CDH.

In our investigations, CRTA values ranged from 254 to 3,225 mm^2^. Dassios et al. (12) reported CRTA values between 1,000 and 2,000 mm^2^ in their collective, and Dimitriou et al. (11) published values from 630 to 1,860 mm^2^. Thus, the values reported are in a similar range. However, it is noticeable that the maximum and minimum values diverge more strongly in our results. Compared with the other studies, a broader spectrum of severity of CDH seems to be represented in our patient cohort.

We additionally defined cutoff values of CRTA related to the three prognostic endpoints. As far as we know, Dassios et al. (12) were the only ones who also defined cutoff values for CRTA, which was 1,299 mm^2^ for survival. It is noticeable that the value for mortality in the current study is lower with 806 mm^2^. A possible explanation for this could be the more heterogeneous patient population in our study. The difference between the cutoff values indicates that further investigation in a larger and multicentric patient collective is necessary before a clinical application of the cutoff values can take place.

The CRTA seems to have an increased prognostic accuracy compared with the prenatally measured and well-established O/E LHR in our study cohort when comparing the AUC values (AUC_survival_ 0.822 vs. 0.674, AUC_ECMO_ 0.802 vs. 0.678, AUC_CLD_ 0.855 vs. 0.700). The value of prognostic accuracy of the O/E LHR differs between studies. For example, for survival, AUC values from 0.732 to 0.782 have been reported from us and others (9, 16–18). For ECMO therapy, we have earlier demonstrated an AUC of 0.612, and for CLD, 0.706 (9).

One limitation of the O/E LHR is the decreased reliability in children with CDH (17). In addition, the ipsilateral lung is not included in measurements. Although it is known that measured O/E LHR correlates with magnetic resonance imaging (MRI) tomographically measured lung volume, the differences are particularly determined by different sized ipsilateral lung volumes (19). This potential underestimation of lung volume via the O/E LHR is even strengthened by the fact that the contralateral lung is more strongly compromised laterally than coronally. Therefore, apical and basal lung volume can be underrepresented by the O/E LHR (20). With prenatal MRI and measured rLV, these limitations can be overcome, and excellent prognostic accuracy for survival (AUC = 0.775), ECMO requirement (AUC = 0.741), and CLD (AUC = 0.792) has been reported. Despite these known limitations of the O/E LHR, it is a broadly used and well-established ultrasound parameter, which is why we chose it as comparison for prognostic accuracy to the CRTA.

As the O/E LHR is determined prenatally and the CRTA on day 1 postnatally, both parameters should not be regarded as competing but as complementary. The CRTA can be measured easily and quickly (approximately 1-min duration) for each child with CDH. Especially for neonates with unknown diagnosis of CDH prenatally, the CRTA seems to be a helpful tool in the prognostic assessment.

One limitation of our study is the monocentric approach of data collection. Future work will have to evaluate whether calculated cutoff values can be transferred to other centers. Another weakness of the present study is that only children with isolated congenital diaphragmatic have been included into analysis to avoid a bias, and therefore, the CRTA has not been evaluated for children with multiple malformations. Future studies should evaluate whether the CRTA is also of prognostic value in this cohort. In addition, in order to recruit a representative and large study cohort, the observation period was quite long (2013–2019). A change in postnatal management, as introduced 2015 (3), might potentially have influenced the outcome parameters and, consequently, the cutoff values of analysis.

## Conclusions

The CRTA can be easily measured in the postnatally acquired chest x-ray for each child with CDH. Despite its simplicity, it shows an excellent prognostic accuracy for important outcome parameters and should therefore be additionally used in the prognostic assessment of children with CDH.

## Data Availability Statement

The original contributions presented in the study are included in the article/Supplementary Material, further inquiries can be directed to the corresponding author.

## Ethics Statement

The studies involving human participants were reviewed and approved by Ethics Committee II of the Medical Faculty Mannheim of the University of Heidelberg (reference number: 2020-802R). Written informed consent from the participants' legal guardian/next of kin was not required to participate in this study in accordance with the national legislation and the institutional requirements.

## Author Contributions

MW, SB, TS, and NR contributed to the concept and design, acquisition, interpretation of data, and drafting of the article. AP, ON, SH, KZ, and SS contributed to the interpretation of data and revised the article for important intellectual content. All authors approved the final version of the article.

## Conflict of Interest

The authors declare that the research was conducted in the absence of any commercial or financial relationships that could be construed as a potential conflict of interest.

## Publisher's Note

All claims expressed in this article are solely those of the authors and do not necessarily represent those of their affiliated organizations, or those of the publisher, the editors and the reviewers. Any product that may be evaluated in this article, or claim that may be made by its manufacturer, is not guaranteed or endorsed by the publisher.
