# Supplementary material for: The Chest Radiographic Thoracic Area Can Serve as a Prediction Marker for Morbidity and Mortality in Infants With Congenital Diaphragmatic Hernia
Source: Front Pediatr. 2021 Dec 23;9:740941. doi: 10.3389/fped.2021.740941 (PMC8733701; doi:10.3389/fped.2021.740941)
Supplement: Supplementary file 1 [file Data_Sheet_1.PDF]

Supplementary Material

1 Supplementary Tables

Suppl. Table 1. Prognostic validity of the observed-to-expected lung-to-head ratio and CRTA for survival considering the side of CDH

	survived	deceased	p-value	AUC	cut-off	sensitivity (%)	specificity (%)
**O/E LHR**							
total	39 (21-83)	33 (21-70)	0.002	0.674	≤36.0	63	67
	(n=137)	*(n=33)*					
left-sided	38.2 (21-83)	31.9 (21-70)	0.003	0.681	≤41.9	41	1
	(n=119)	*(n=30)*					
right-sided	42.9 (29-78)	46 (33-52)	0.804	0.556	≤56.1	0	1
	(n=18)	*(n=3)*					
**CRTA**							
total	1252 (434-3225)	682 (254-1919)	<0.0001	0.822	≤806	88	69
	(n=213)	*(n=42)*					
left-sided	1295 (434-3225)	682 (254-1919)	<0.0001	0.818	≤809	89	67
	(n=186)	*(n=39)*					
right-sided	1048 (615-2552)	497 (332-736)	0.022	0.938	≤751	82	1
	(n=27)	*(n=3)*					

Data are expressed as median (interquartile range) for O/E LHR and in mm^2 for CRTA.
AUC, are under curve; *CDH*, congenital diaphragmatic hernia; *CRTA*, chest radiographic thoracic area; *LHR*, lung-to-head ratio; *O/E*, observed-to-expected

Suppl. Table 2. Prognostic validity of the observed-to-expected lung-to-head ratio and CRTA for ECMO support considering the side of CDH

	no ECMO	ECMO	p-value	AUC	cut-off	sensitivity (%)	specificity (%)
**O/E LHR**							
total	42.5 (23-83) *(n=80)*	34.6 (21-78) *(n=90)*	<0.0001	0.678	≤39	69	61
left-sided	42 (23-83) *(n=77)*	32 (21-70) *(n=72)*	<0.0001	0.728	≤39	78	60
right-sided	56 (41-63) *(n=3)*	42.9 (29-78) *(n=18)*	0.376	0.676	≤52	78	67
**CRTA**							
total	1470 (437-3225) *(n=137)*	875 (254-2258) *(n=118)*	<0.0001	0.802	≤802	78	70
left-sided	1462 (437-3225) *(n=130)*	874 (254-2258) *(n=95)*	<0.0001	0.797	≤797	78	69
right-sided	1596 (852-2552) *(n=7)*	876 (332-1875) *(n=23)*	0.008	0.863	≤863	83	86

Data are expressed as median (interquartile range) for O/E LHR and in mm^2 for CRTA.
AUC, are under curve; *CDH*, congenital diaphragmatic hernia; *CRTA*, chest radiographic thoracic area; *ECMO*, extracorporeal membrane oxygenation; *LHR*, lung-to-head ratio; *O/E*, observed-to-expected

Suppl. Table 3. Prognostic validity of the observed-to-expected lung-to-head ratio and CRTA for chronic lung disease considering the side of CDH

	no CLD	CLD	p-value	AUC	cut-off	sensitivity (%)	specificity (%)
**O/E LHR**							
total	45 (27-74)	36 (21-78)	0.0002	0.700	≤39	68	71
	(n=51)	*(n=81)*					
left-sided	43.9 (27-74)	34.4 (21-70)	<0.0001	0.723	≤39	76	69
	(n=48)	*(n=66)*					
right-sided	63 (56-65.4)	41 (29-78)	0.093	0.844	≤47	1?	1?
	(n=3)	*(n=15)*					
**CRTA**							
total	1610 (787-3225)	997 (434-2379)	<0.0001	0.855	≤855	86	74
	(n=91)	*(n=117)*					
left-sided	1617 (787-3225)	1013 (434-2379)	<0.0001	0.848	≤848	86	73
	(n=84)	*(n=97)*					
right-sided	1596 (852-2552)	919 (615-1875)	0.007	0.879	≤879	1	86
	(n=7)	*(n=20)*					

Data are expressed as median (interquartile range) for O/E LHR and in mm^2 for CRTA.
AUC, are under curve; *CDH*, congenital diaphragmatic hernia; *CLD*, chronic lung disease; *CRTA*, chest radiographic thoracic area; *LHR*, lung-to-head ratio; *O/E*, observed-to-expected

Supplementary Figures

Suppl. Figure 1.

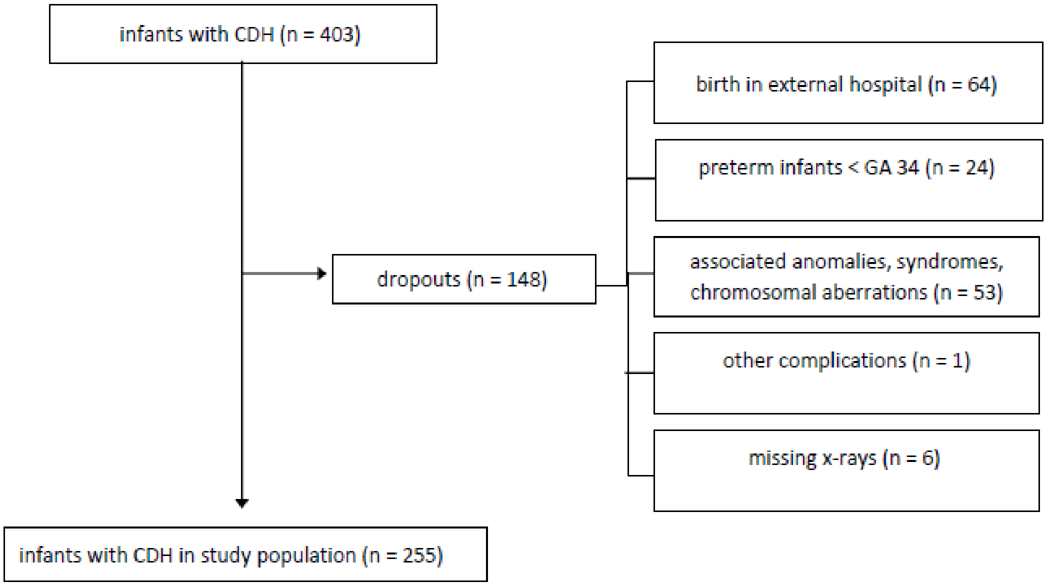

	died	survived	total
CLD	6	111	117
no CLD	36[a]	102[b]	138
total	42	213	255

Suppl. Figure 2.

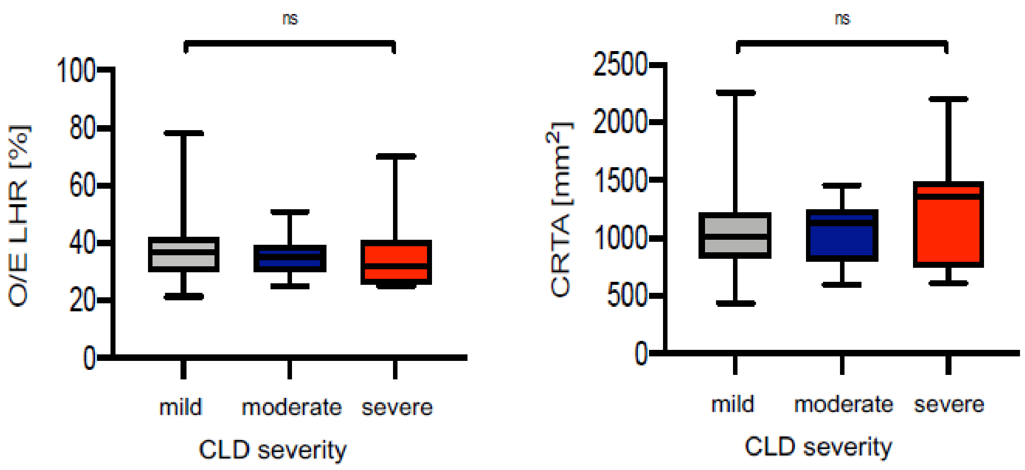

Suppl. Figure 3.

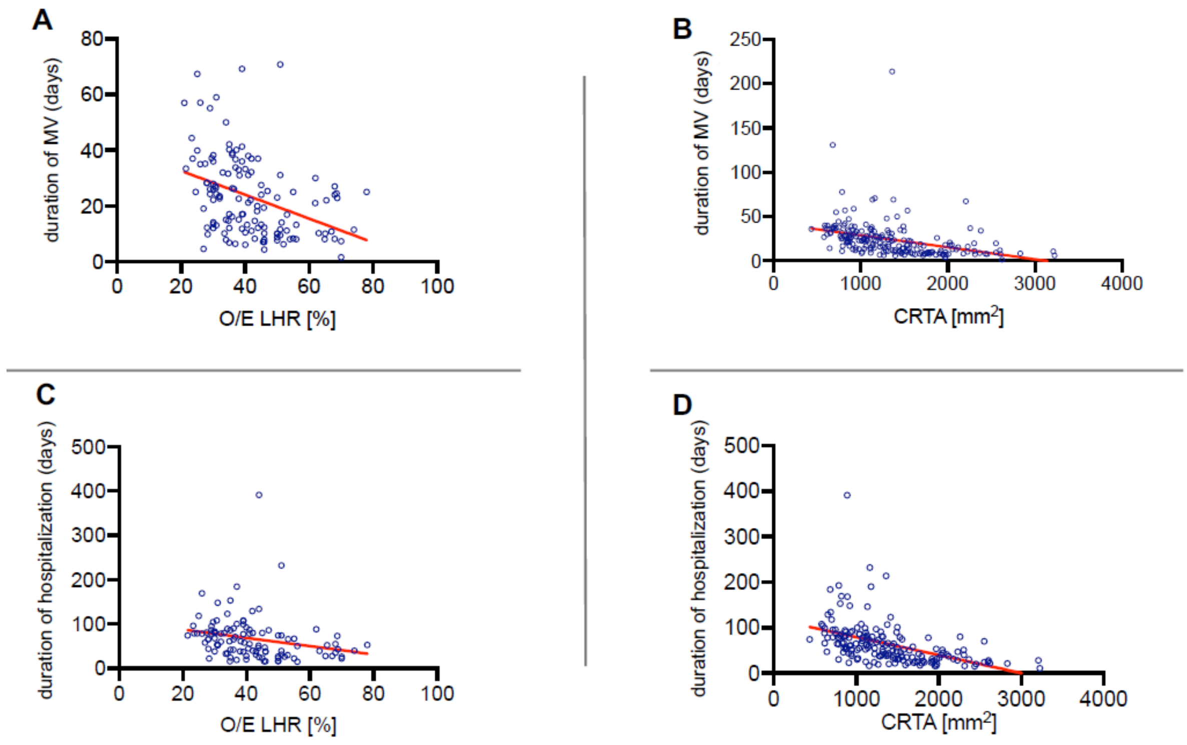

Figure Legends

Supplementary Figure 1: Flow chart for recruitment of newborn with CDH in our study.
[a]Infants who died before day 28 of life were excluded from CLD analysis, since diagnosis of CLD is assessed on day 28. [b]Infants who were transferred to an external hospital with oxygen therapy or ventilatory support before day 28 of life were excluded from CLD analysis, since diagnosis of CLD is assessed on day 28.
CDH, congenital diaphragmatic hernia; *CLD*, chronic lung disease; *GA*, gestagional age.

Supplementary Figure 2: Correlation of observed-to-expected lung-to-head ratio and CTRA to CLD severity. Boxplots of **(A)** observed-to-expected lung-to-head ratio and (**B**) CRTA in CDH infants with mild, moderate and severe CLD (whiskers are shown as minimum and maximum). [ns] represents a non-significant difference in the Kruskal-Wallis test (O/E LHR: p = 0.778; CRTA: p = 0.663).
CLD, chronc lung disease; *CRTA*, chest radiographic thoracic area; *LHR*, lung-to-head-ratio; *O/E*, observed-to-expected.

Supplementary Figure 3: Correlation of observed-to-expected lung-to-head ratio and CTRA to duration of mechanical ventilation and of hospitalization.
Scatterplots portraying the correlation of observed-to-expected lung-to-head ratio and CRTA to the duration of mechanical ventilation (A and B) and the duration of hospitalization (C and D).
CRTA, chest radiographic thoracic area*;* *LHR*, lung-to-head-ratio; *MV*, mechanical ventilation; *O/E*, observed-to-expected.
